# Management of plastic bronchitis with nebulized tissue plasminogen activator: another brick in the wall

**DOI:** 10.1186/1824-7288-40-18

**Published:** 2014-02-13

**Authors:** Massimo Colaneri, Andrea Quarti, Marco Pozzi, Stefano Gasparini, Ines Carloni, Fernando Maria de Benedictis

**Affiliations:** 1 Department of Cardiovascular Medicine, Azienda Ospedaliero-Universitaria Ospedali Riuniti, Ancona, Italy; 2 Department of Internal Medicine, Azienda Ospedaliero-Universitaria Ospedali Riuniti, Ancona, Italy; 3 Department of Mother and Child Health, Salesi Children’s Hospital, Azienda Ospedaliero-Universitaria Ospedali Riuniti, Ancona, Italy

**Keywords:** Plastic bronchitis, Tissue plasminogen activator, Primary ciliary dyskinesia

## Abstract

Plastic bronchitis is a rare complication of a variety of respiratory diseases and congenital heart disease surgery, particularly Fontan procedure. Bronchial casts with rubber-like consistency develop acutely and may cause severe life-threatening respiratory distress. The management of plastic bronchitis is yet not well defined. Early intermittent, self-administered nebulization of tissue plasminogen activator was found to be effective in preventing deterioration of acute respiratory symptoms in a patient with primary ciliary dyskinesia and recurrent cast formation. Further investigation into new therapeutic strategies for this devastating disease is advocated.

## Background

Plastic bronchitis (PB) is an uncommon condition characterized by mucoid impaction with casts that occlude major bronchi and cause acute, potentially fatal bronchial obstruction [1]. PB has been associated with a variety of respiratory diseases, but is found most commonly as a secondary phenomenon after Fontan surgery in patients with cyanotic congenital heart disease (CHD) [2]. Especially in these patients, recurrences are common and mortality remains high. Patients either expectorate casts spontaneously, coughing up a firm mucus material, or may require urgent bronchoscopy to remove casts and avoid impending respiratory arrest due to airway obstruction.

The pathophysiology of PB remains to be elucidated. It has been suggested that high pulmonary venous pressures may lead to an abnormal response of respiratory epithelium and/or lymphatic dysfunction, thus resulting in excess mucus production and cast formation. An underlying genetic predisposition associated with an inflammatory trigger was also postulated to explain the abnormal mucin hypersecretion [3]. PB has been classified in type 1 (inflammatory) and 2 (noninflammatory) according to the characteristics of casts. Because inflammation is thought to play a role in the formation of all casts, Madsen et al. [3] have recommended classification based on associated disease first and on cast histology second.

We report the case of an adolescent with CHD and primary ciliary dyskinesia (PCD) who developed features of PB and severe respiratory failure 7 yrs apart from Fontan operation. The occurrence of PB in our patient let us hypothesize that decreased mucociliary clearance was an adjunctive trigger for the development of PB. The effect of early, intermittent nebulization of recombinant human tissue plasminogen activator (t-PA) therapy is described.

## Case presentation

A 13-year-old boy presented with a history of 10-day fever, cough, and progressive dyspnea which persisted despite antibiotic treatment. He was born with right atrial isomerism, situs viscerum inversus, asplenia and complex CHD characterized by double outlet right ventricle, great arteries transposition, pulmonary artery stenosis, ventricular septal defect and common atrio-ventricular valve. At age 2 yrs, PCD was diagnosed by electron microscope specimen examination of respiratory cilia (100 percent of abnormal cilia with both inner and outer dynein arm defect). Glenn operation and fenestrated extracardiac Fontan surgery were performed at age 3 yrs and 6 yrs, respectively. Over the years the patient developed chronic oto-sino-pulmonary disease. For recurrent wheezing and productive cough with abundant, thick expectoration, regular antiasthmatic treatment and chest physiotherapy was instituted.

**Figure 1:**
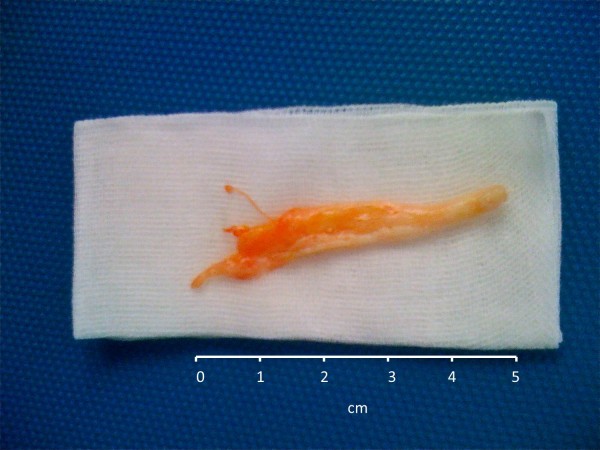
Large mucinous cast removed by rigid bronchoscopy.

At admission, physical examination revealed a child in severe respiratory distress; scattered, harsh breath sounds were present at chest auscultation. Blood pressure was 50/40 mmHg. Arterial blood gas analysis showed pH 6.9, PaO2 61 mmHg, SaO2 88%, PaCO2 150 mmHg. The patient was immediately intubated and mechanically ventilated, and was admitted to the Intensive Care Unit. Chest radiography revealed patchy consolidation in the right middle lobe and mild pleural effusion. Echocardiography showed an ejection fraction of 55% and moderately dilated inferior vena cava (IVC) and superior vena cava (SVC), with normal flow and low blood velocity. A low cardiac output syndrome occurred with multiorgan failure, requiring a continuous veno-venous ultrafiltration and pharmacologic support, including intravenous dopamine, epinephrine and enoximone. Cardiac catheterization showed satisfactory hemodynamics with no obstruction of the Fontan circulation; IVC and SVC pressure was 21 mmHg; total pulmonary resistance and arteriolar pulmonary resistance were 3,8 U/m2 and 1,5 U/m2, respectively. The QP/QS ratio was 0,9 and no stenosis at the anastomotic site was demonstrated. A flexible fiber optic bronchoscopy revealed large, mucinous casts partially obstructing the airway tree. Rigid bronchoscopy was required to remove the casts (Figure 1). An immediate and sustained improvement in oxygenation was obtained. Histological examination revealed mostly mucous material with fibrin and rare inflammatory cells. Cultures taken from the casts were negative. The patient was extubated on hospital day 12 and was maintained on therapy with albuterol, N-acetylcysteine, budesonide, deoxyribonuclease (DNase) by nebulization, and oral sildenafil. Despite such therapy, other two episodes of respiratory deterioration occurred over the following 10 days, each requiring bronchoscopic removal of casts from the bronchial tree. After the latest episode, treatment with aerosolized t-PA (Actilyse, Boehringer Ingelheim) was added to the previous therapy. A total of 5 ml of the drug diluted to 1 mg/ml of normal saline was delivered every 6 hours via a Pari TurboBoy S compressor with a Pari LC Sprint reusable nebulizer and a mask (Pari GmbH, Starnberg, Germany). Over the next few days, the child started expectorating thinner bronchial secretions and his condition improved gradually, with clearing of areas of atelectasis and no recurrence of respiratory symptoms. The patient was discharged home receiving budesonide (0,5 mg twice daily), DNase (1,25 mg twice daily) and t-PA (5 mg four times daily) by nebulization, oral sildenafil, and chest physiotherapy.

During an elective admission after 2 months, an attempt at gradually suspending t-PA treatment was successful. At discharge, parents received instructions to reintroduce a course of t-PA at the onset of symptoms or signs (cough, expectoration or chest discomfort) that they identified as their child’s usual starting point before the development of PB. T-PA starting dose of 5 mg had to be repeated after 15 and 60 min, and then continued four times daily for 7 days or until symptoms had completely resolved. Parents were also instructed to refer immediately the child to the hospital if symptoms had not improved after the first three doses, and to regularly contact the medical staff within 24 hours after starting treatment in order to report the event. During 12 month follow-up, this therapeutic regimen had to be reintroduced eight times, when respiratory symptoms suggested an impending risk of cast formation. Prompt and complete relief was obtained in each occasion, with no side effects and no further hospital admissions.

## Discussion

Patients with CHD who received Fontan operation have more life-threatening events and a greatest mortality rate due to episodes of PB compared with those with other underlying diseases [4]. Preventing recurrence of PB is therefore a major challenge for patients with Fontan physiology. Optimization of cardiac output and consideration of low-fat diet or thoracic duct ligation are important therapeutic measures. The indication for heart transplantation remains uncertain, particularly in patients who improve on full medical treatment.

Over the years, strategies to enhance cast expectoration or to induce lysis of casts have been repeatedly claimed. Several drugs for the treatment of PB have been used, including antibiotics, steroids, bronchodilators, diuretics, unfractionated heparin, N-acetylcysteine, urokinase, DNase and t-PA [1]. However, most of the evidence on the effect of pharmacologic therapy comes from individual case reports or small case series that do not allow to compare the efficacy of a drug against the others. Furthermore, most patients received a number of different medications making it difficult to ascertain which of them is really effective.

We believe that our case is worth mentioning for several reasons. First, a long delay in presenting symptoms of PB after Fontan surgery is unusual [5], as the onset of PB is typically within 1 to 3 years after surgical operation. This finding alerts to regularly evaluate cardiac function and watch over the symptoms of PB in patients who received Fontan intervention. Second, only one case of PB in Kartagener’s syndrome has been reported so far, and the therapeutic approach after emergency removing of bronchial casts was not described [6]. It is likely that underlying immotile cilia dysfunction and decreased mucociliary clearance may influence the development of recurrent bronchial casts in patients with associated PCD and CHD who received Fontan circulation. Third, we first report on the efficacy of intermittent use of aerosolized t-PA in preventing recurrence of PB. Given the severity of symptoms before starting t-PA, the improvement observed in our patient was a likely consequence of treatment and not a simple matter of time.

T-PA is a serine protease which exerts the fibrinolytic action by catalyzing the conversion of plasminogen to plasmin. Quasney et al. [7] first demonstrated that bronchial casts were unchanged with in vitro saline incubation, became soft and friable when incubated with urokinase, but dissolved completely when incubated in a t-PA solution. Gibb et al. [8] first demonstrated that direct instillation of t-PA into the airways during bronchoscopy was an effective therapy for PB, being superior to nebulized DNase. A recent retrospective study reported that casts in patients with CHD and Fontan physiology are predominantly hypocellular and fibrinous, thus substantiating the role of local t-PA therapy [9].

There have been several reports on the effect of inhaled t-PA in improving PB. However, the optimal dosing strategy and the duration of therapy remain unclear. Repeated doses up to 2 mg/kg per day have been used with success without important side effects [4]. In a ex vivo study, increasing amounts of aerosolized t-PA did not improve cast weight reduction and no evidence of dose–response effect was found using this endpoint [10]. Furthermore, 28-day repeated administration of high doses (>1 mg/kg/day) inhaled t-PA in mice resulted in pulmonary haemorrhage, whereas low-dose therapy was well tolerated [11]; it was supposed that this side effect may be the consequence of the drug accumulation in the lungs. Since rigorous information on the optimal t-PA dosage is lacking, low doses (0.5 mg/kg) have been used in our experimental protocol. We cannot however exclude that higher doses may be more effective, especially if casts are refractory to treatment.

Should t-PA therapy be administered on continuous basis to prevent cast formation? The actual experience is largely anecdotal. In a patient who had failed several attempts to stop treatment, long-term administration of aerosolized t-PA was highly effective to avoid recurrent episodes of life-threatening airway obstruction, and was safe [12]. Regular nebulization of t-PA has been recently included into a multi-drug protocol of medical treatment, but the results are limited to less than 1 year follow-up [4]. Interestingly, a new strategy on alternate weeks t-PA administration led to no recurrence of cast formation in a 4-year old boy with Fontan circulation [13].

T-PA is extremely expensive. We speculated that early, intermittent nebulization of this drug during a pre-defined acute episode instead of regular, “preventive” administration may avoid the progression of respiratory symptoms and reduce costs. This strategy was effective and safe in our patient. As vain attempts to wean patients with recurrent PB off regular therapy with t-PA have been described [12,13], it seems however prudent to taper the treatment gradually under close medical supervision. Whether regular t-PA administration is more effective than intermittent strategy needs to be ascertained in future studies.

In conclusion, prompt self-administration of t-PA avoided the recurrence of cast formation in a child with PCD and Fontan physiology. Given our single case experience, such strategy should be limited to patients who were able to interrupt regular treatment with t-PA with no deterioration. Characterizing t-PA distribution in the airway in order to optimize the dose delivered to the lungs, understanding whether patients with particular casts histology (i.e. high fibrin concentration) may better benefit by t-PA inhalation and assessing the role of combined therapies represent some key points to be urgently identified. An International Plastic Bronchitis registry to collect data on patients worldwide (http://www.clinicaltrials.gov, NCT01663948) has been recently established with the aim to answer still unresolved questions for this devastating disease [14].

## Consent

Written informed consent was obtained from the patient’s parents for publication of this case report and any accompanying images. A copy of written consent is available for review by the editor-in-chief of this journal.

## Abbreviations

PB: Plastic bronchitis; CHD: Congenital heart disease; t-PA: Tissue plasminogen activator; IVC: Inferior vena cava (IVC); SVC: Superior vena cava (SVC); DNase: Deoxyribonuclease.

## Competing interests

The authors declare that they have no competing interests.

## Authors’ contributions

MC identified the case, suggested the diagnosis and drafted the manuscript. AQ and MP were involved in the diagnostic process, therapeutic decisions and follow up. IC helped in writing the manuscript and selecting bibliography. SG made substantial contribution to bronchoscopy studies. FMdB supervised the diagnostic and therapeutic approach, and critically revised the manuscript. All authors read and approved the final manuscript.
